# Susceptibility patterns of *Staphylococcus aureus* biofilms in diabetic foot infections

**DOI:** 10.1186/s12866-016-0737-0

**Published:** 2016-06-23

**Authors:** Carla Mottola, Carina S. Matias, João J. Mendes, José Melo-Cristino, Luís Tavares, Patrícia Cavaco-Silva, Manuela Oliveira

**Affiliations:** Centro de Investigação Interdisciplinar em Sanidade Animal (CIISA), Faculdade de Medicina Veterinária, Universidade de Lisboa, Avenida da Universidade Técnica, 1300-477 Lisbon, Portugal; Departamento de Medicina Interna, Hospital de Santa Marta/Centro Hospitalar de Lisboa Central, EPE, Lisbon, Portugal; Faculdade de Medicina, Universidade de Lisboa, Instituto de Microbiologia, Lisbon, Portugal; TechnoPhage, S.A., Lisbon, Portugal; Centro de Investigação Interdisciplinar Egas Moniz (CiiEM), Instituto Superior de Ciências da Saúde Egas Moniz, Monte de Caparica, Portugal

**Keywords:** *Staphylococcus aureus*, Diabetic foot infections, MBIC, MBEC, Resistance genes

## Abstract

**Background:**

Foot infections are a major cause of morbidity in people with diabetes and the most common cause of diabetes-related hospitalization and lower extremity amputation. *Staphylococcus aureus* is by far the most frequent species isolated from these infections. In particular, methicillin-resistant *S. aureus* (MRSA) has emerged as a major clinical and epidemiological problem in hospitals. MRSA strains have the ability to be resistant to most β-lactam antibiotics, but also to a wide range of other antimicrobials, making infections difficult to manage and very costly to treat. To date, there are two fifth-generation cephalosporins generally efficacious against MRSA, ceftaroline and ceftobripole, sharing a similar spectrum.

Biofilm formation is one of the most important virulence traits of *S. aureus.* Biofilm growth plays an important role during infection by providing defence against several antagonistic mechanisms. In this study, we analysed the antimicrobial susceptibility patterns of biofilm-producing *S. aureus* strains isolated from diabetic foot infections. The antibiotic minimum inhibitory concentration (MIC) was determined for ten antimicrobial compounds, along with the minimum biofilm inhibitory concentration (MBIC) and minimum biofilm eradication concentration (MBEC), followed by PCR identification of genetic determinants of biofilm production and antimicrobial resistance.

**Results:**

Results demonstrate that very high concentrations of the most used antibiotics in treating diabetic foot infections (DFI) are required to inhibit *S. aureus* biofilms in vitro, which may explain why monotherapy with these agents frequently fails to eradicate biofilm infections. In fact, biofilms were resistant to antibiotics at concentrations 10–1000 times greater than the ones required to kill free-living or planktonic cells. The only antibiotics able to inhibit biofilm eradication on 50 % of isolates were ceftaroline and gentamicin.

**Conclusions:**

The results suggest that the antibiotic susceptibility patterns cannot be applied to biofilm established infections. Selection of antimicrobial therapy is a critical step in DFI and should aim at overcoming biofilm disease in order to optimize the outcomes of this complex pathology.

## Background

Foot infections are a major cause of morbidity in diabetes patients and the most common cause of diabetes-related hospitalization and lower limb amputation [1]. The physiopathology of diabetic foot infections (DFI) is complex, but its severity and prevalence are a consequence of host-related disorders and pathogens-factors, as virulence and antibiotic resistance traits [1]. DFI are mostly polymicrobial and *Staphylococcus aureus* is by far the more frequent species involved, either alone or as a component of mixed infections [2, 3].

*S. aureus* is an important nosocomial pathogen that can cause several infections such as: bacteraemia, osteomyelitis, skin infections, pneumonia, meningitis and endocarditis. In particular, MRSA has emerged as a major clinical and epidemiological problem in hospitals since the 1980s [4]. MRSA strains have the ability to resist to all β-lactam antibiotics and also to a wide range of other antimicrobials, making MRSA infections costly and difficult to manage [5]. Ceftaroline and ceftobiprole are, to date, the only anti-MRSA cephalosporins that inhibit PBP2a (penicillin binding protein 2a) at therapeutically concentrations. Ceftobiprole, already evaluated in clinical trials, access the active site of PBP2a by its R2 group, whereas ceftaroline causes an allosteric change in PBP2a [6]. Ceftaroline is FDA approved for treatment of skin and skin structure infections, including those caused by MRSA [6].

Several structural and secreted virulence factors play a role in *S. aureus* infections, which are multifactorial and depend on bacterial adherence and biofilm formation. In the beginning of an infection, *S. aureus* produces numerous surface proteins, called “microbial surface components recognizing adhesive matrix molecules” (MSCRAMM) that mediate adherence to host tissues. Once *S. aureus* adheres to host tissues, it can form biofilms, which enable its persistence by allowing bacteria to evade host defences, impeding access to certain types of immune cells, such as macrophages, which display incomplete penetration into the biofilm matrix and “frustrated phagocytosis” [7]. Additionally, biofilm cells display increased tolerance to antibiotics [8].

In contrast to heritable antibiotic resistance mechanisms, biofilm-associated tolerance is a transient state in which normally susceptible bacteria display an altered physiology that decreases sensitivity. When these cells disperse and re-enter in a planktonic state, they present their normal susceptibility profile [9]. Bacteria embedded within a biofilm are difficult to eradicate due to a wide variation of nutrient gradients that slow or arrest bacterial growth, protein synthesis, and other physiologic activities; bacteria sequestered in biofilms are less susceptible to antibiotics by virtue of their reduced growth rates [10]. Other factors that contribute to biofilm-mediated antimicrobial resistance include inefficient diffusion or sequestering of the agent within biofilm matrix, the presence of “persister” cells and other unknown phenotypic differences [10].

Various genes have been implicated in the onset and maintenance of biofilms by staphylococci. Among these, the most extensively studied are *icaA* and *icaD* (intercellular adhesion A and B), products of a gene locus composed by the genes *icaR* (intercellular adhesion regulator) and *ica A, B, C,* and *D* (intercellular adhesion ABCD), responsible for the synthesis of polysaccharide intercellular adhesin (PIA), which contains N-acetylglucosamine, a major component of the exopolysaccharide matrix that surrounds bacterial cells in the biofilm [11]. Also, the products of *pls* (plasmin sensitive) which encodes a surface protein, and *atl* (autolysin) which encodes an autolysin, have been implicated in the formation and structuring of biofilms. The *atl* is the most predominant peptidoglycan hydrolase in staphylococci, and was also identified as an adhesin involved in primary attachment of cells to polystyrene surfaces [12]. The *pls* is a homologue of the serine-aspartate repeat (Sdr) surface protein family, of which *ClfA* (clumping factor A) is the best-characterized member, that reduces adhesion to host proteins and cellular invasiveness [13].

In this study, a collection of *S. aureus* strains isolated from DFI was characterized in terms of their planktonic and biofilm susceptibility patterns, and presence of biofilm and antibiotic resistance genes. The antibiotic minimum inhibitory concentration (MIC) was determined, along with the minimum biofilm inhibitory concentration (MBIC) and minimum biofilm eradication concentration (MBEC), followed by PCR identification of genetic determinants of biofilm production and antimicrobial resistance.

## Methods

### Strains

A total of 53 staphylococci clinical isolates from diabetic foot ulcers (DFU), obtained from 49 samples, were collected in a previous epidemiological survey, as described by Mendes et al. in 2012 [2]. From this collection, twenty-three (*n* = 23) representative biofilm-producing *S. aureus* isolates were selected, based on Pulse Field Gel Electrophoresis (PFGE) and Multilocus Sequence Type (MLST) analysis, previously performed by our research team [14]. A reference strain, *S. aureus* ATCC 29213, a known biofilm producer, was also included in this study.

### Antimicrobial agents

The antibiotics cefoxitin (FOX), ciprofloxacin (CIP), clindamycin (CLI), doxycycline (DOX), erythromycin (ERY), gentamicin (GEN), linezolid (LZD), meropenem (MEM) and vancomycin (VAN) were obtained from Sigma-Aldrich (Portugal). AstraZeneca (Portugal) generously provided ceftaroline (CPT). All antibiotics were prepared according to CLSI guidelines [15].

### Minimum inhibitory concentrations

MIC were determined for all antibiotics to all strains; e-test was performed according to the manufacturer’s recommendations (Biomérieux). Test performance was monitored using the reference strain *S. aureus* ATCC 29213.

### Biofilm susceptibility tests

A modified version of the Calgary Biofilm Pin Lid Device (CBPD) [16] was used to determine the antimicrobial susceptibility of bacteria embedded in a 24-h biofilm, in order to determine the MBIC and MBEC [16, 17]. Briefly, a starting inoculum of 5×10^5^ CFU/mL in Mueller Hinton Broth (MHB, Liofilchem Italy) was distributed in 96-well flat-bottom microtiter plates (Nunc, Roskilde, Denmark), covered with a 96-peg lid (Imuno TSP; Nunc, Roskilde, Denmark) and statically incubated for 24-h at 35 °C, to allow biofilm formation on the pegs [18, 19]. The peg lid was then rinsed three times in 1X sterile PBS to remove planktonic bacteria, placed on a new plate filled with 200 μL of fresh broth containing serial dilutions of antibiotics, from 1024 μg/ml to 0.5 μg/ml, and incubated for 24-h at 37 °C [17–20]. After incubation, the peg lid was removed and the MBIC value was recorded and defined as the last well in which there was no visible growth after incubation [17, 21]. Next, to determine the MBEC value, the peg lid was rinsed three times in 1X sterile PBS, placed in a new plate filled with 200 μL of fresh MHB and sonicated at 45–60 Hz during 10 min [17, 22], in order to disperse the bacteria from the peg surface. After sonication, the peg lid was discarded and the plate was covered with a normal lid and incubated for 24-h at 37 °C. After incubation, the quantification of biofilm formation was conducted according with a previously described colorimetric microtiter plate, using Alamar Blue [23]. Briefly, 5 μl of resazurin (Alamar Blue, AB, ThermoScientific, Spain) was added to the wells and the plates were incubated for one hour at 37 °C. After incubation, absorbance (A) values at 570 nm and 600 nm were recorded in a microplate reader (BMG LABTECH GmbH, Germany). Controls included media plus Alamar Blue (control one) and bacterial cells plus media plus AB without antibiotic (control two). The MBEC was defined as the lowest drug concentration resulting in ≤1/2 the absorbance value when compared to control two. Assays were performed at least twice and the average absorbance values used to determine the MBEC values.

### PCR screening of biofilm associated and antibiotic resistance genes

All strains were investigated to detect the presence of genes associated with biofilm formation, namely: *icaA*, *icaD*, *atl* and *pls*. Genes associated with antibiotic resistance were also screened, namely: *blaZ* (penicillin resistance); *mecA* and *mecC* (oxacillin resistance); *tetK*, *tetL*, *tetM* and *tetO* (tetracycline resistance); *msrA*, *ermA*, *ermB* and *ermC* (erythromycin resistance); *aac(6′)-aph(2″)* (gentamicin resistance) and *norA* (ciprofloxacin resistance).

Detection of *mecA* gene and its homologous *mecC* were performed by multiplex PCR [24]. Oligonucleotide primer sequences are described in Table 1. All amplification reactions were prepared with a mixture containing: 12.5 μl of Supreme NZYTaq 2x Green Master Mix (NZYTech, Portugal), 1 μl of each primer (forward and reverse) (STABVIDA Lda, Portugal) and 5.5 μl of sterile water (water for molecular biology, NZYTech, Portugal). To this mixture, 1 μl of the previous extracted DNA was added, resulting in a total reaction volume of 25 μl. PCR amplification was performed in a thermal cycler (MyCycler Thermal Cycler, Bio-Rad, Portugal) using conditions described in the references reported in Table 1. Positive controls for the tested genes were gently provided by: Dr. Mark Holmes (University of Cambridge, England), Dr. Penadés (Cardenal Herrera University, Valencia, Spain) and Dr. Birgit Strommenger (Robert Koch Institute, Berlin, Germany).

**Table 1:** PCR target genes and primers used in this work

Gene	Primer	Reference
	Sequence (5′→3′)	
*icaA*	TCTCTTGCAGGAGCAATCAA	Arciola et al. (2001) [46]
	AGGCACTAACATCCAGCA	
*icaD*	ATGGTCAAGCCCAGACAGAG	Arciola et al. (2001) [46]
	CGTGTTTTCAACATTTAATGCAA	
*atl*	CTTCAGCACAACCAAGATC	Petrelli et al. (2008) [4]
	GGTTACCGACTGCACCGTCAC	
*pls*	GTAATACAACAGGAGCAGATGG	Petrelli et al. (2008) [4]
	GTAGCTTTCCATGTTTTTCCTG	
*blaZ*	ACTTCAACACCTGCTGCTTTC	Martineau et al. (2000) [38]
	TGACCACTTTTATCAGCAACC	
*mecA*	TCCAGATTACAACTTCACCAGG	Stegger et al. (2012) [24]
	CCACTTCATATCTTGTAACG	
*mecC*	GAAAAAAAGGCTTAGAACGCCTC	Stegger et al. (2012) [24]
	GAAGATCTTTTCCGTTTTCAGC	
*tetK*	TCGATAGGAACAGCAGTA	Ng et al. (2001) [47]
	CAGCAGATCCTACTCCTT	
*tetL*	TCGTTAGCGTGCTGTCATTC	Ng et al. (2001) [47]
	GTATCCCACCAATGTAGCCG	
*tetM*	GTGGACAAAGGTACAACGAG	Ng et al. (2001) [47]
	CGGTAAAGTTCGTCACACAC	
*tetO*	AACTTAGGCATTCTGGCTCAC	Ng et al. (2001) [47]
	TCCCACTGTTCCATATCGTCA	
*msrA*	TCCAATCATTGCACAAAATC	Martineau et al. (2000) [38]
	AATTCCCTCTATTTGGTGGT	
*ermA*	TATCTTATCGTTGAGAAGGGATT	Martineau et al. (2000) [38]
	CTACACTTGGCTTAGGATGAAA	
*ermB*	CTATCTGATTGTTGAAGAAGGATT	Martineau et al. (2000) [38]
	GTTTACTCTTGGTTTAGGATGAAA	
*ermC*	CTTGTTGATCACGATAATTTCC	Martineau et al. (2000) [38]
	ATCTTTTAGCAAACCCGTATTC	
*aac(6′)–aph(2″)*	TTGGGAAGATGAAGTTTTTAGA	Martineau et al. (2000) [38]
	CCTTTACTCCAATAATTTGGCT	
*norA*	TTCACCAAGCCATCAAAAAG	Pourmand et al. (2014) [48]
	CTTGCCTTTCTCCAGCAATA	

## Results

### Minimum inhibitory concentrations

All isolates were considered susceptible to vancomycin, linezolid and doxycycline, with MIC values ≤1 μg/ml, ≤4 μg/ml and ≤0.5 μg/ml respectively. Ceftaroline MIC values were ≤0.5 μg/ml and only two isolates presented MIC ≥4 μg/ml (ceftaroline-resistant). All isolates, except for one, originated MIC values for clindamycin of ≤0.5 μg/ml. Gentamicin MIC values were ≤1 μg/ml, except for three resistant isolates. About 57 % of isolates were considered susceptible to ciprofloxacin with MIC ≤2 μg/ml, and 65 % were erythromycin susceptible with MIC ≤0.5 μg/ml. Eight isolates (35 %) were cefoxitin-resistant, with MIC values ≥8 μg/ml (Tables 2 and 3).

**Table 2:** In vitro MIC, MBIC and MBEC values for the antibiotics tested against *S. aureus* DFU isolates (*CLSI range susceptibility)

	Antimicrobial agents									
	FOX	CPT	CIP	CLI	DOX	ERY	GEN	LZD	MEM	VAN
MIC range	1.5–256	0.064–38	0.06- >32	0.015–0.06	0.064–0.125	0.12- >256	0.06–64	1–2	0.015–16	0.25–1
MBIC range	2–256	0.5–8	0.5–512	0.5–128	0.5–512	0.5- >256	0.5- >128	1- >1024	0.5–32	1–16
MBEC range	2–1024	0.5–1024	256–512	64- >1024	64–128	64- >1024	1- >256	4- >1024	0.5- >1024	8- >1024

FOX, cefoxitin (≤4 μg/ml*); CPT, ceftaroline (≤0.5 μg/ml *); CIP, ciprofloxacin (≤4 μg/ml *); CLI, clindamycin (≤0.25 μg/ml*); DOX, doxycycline (≤4 μg/ml*); ERY, erythromycin (≤8 μg/ml*); GEN, gentamicin (≤4 μg/ml*); LZD, linezolid (≤4 μg/ml*); MEM, meropenem (≤4 μg/ml*); VAN, vancomycin (≤2 μg/ml*)

**Table 3:** Antibiotic resistance phenotypes and genotypes of *S. aureus* DFU isolates

Isolate	Resistance																						
	Phenotype										Gene												
	FOX	CIP	CPT	CLI	DOX	ERY	GEN	LZD	MEM	VAN	*blaZ*	*mecA*	*mecC*	*ermA*	*ermB*	*ermC*	*msrA*	*norA*	*tetK*	*tetL*	*tetM*	*tetO*	*aac(6′)-aph(2″)*
I01	R	R	S	S	S	S	S	S	I	S	-	+	-	+	-	-	-	+	-	-	-	-	+
I02	S	R	R	S	S	S	S	S	S	S	-	-	-	-	-	-	-	+	-	-	-	-	-
I03	S	S	S	S	S	S	S	S	S	S	-	-	-	-	-	-	-	+	-	-	-	-	-
I04	S	S	S	S	S	S	S	S	S	S	-	-	-	-	-	+	-	+	-	-	-	-	-
I05	S	S	S	S	S	S	S	S	S	S	-	-	-	-	-	-	-	+	-	-	-	-	-
I06	R	R	S	S	S	R	S	S	S	S	-	+	-	+	-	-	-	+	-	-	-	-	-
I07	R	R	S	R	S	R	R	S	R	S	+	+	-	-	-	-	-	+	-	-	+	-	-
I08	R	R	R	S	S	R	S	S	I	S	-	+	-	-	-	+	-	-	-	-	-	-	-
I09	R	R	S	S	S	R	S	S	R	S	-	+	-	-	-	+	-	-	-	-	-	-	-
I10	S	R	S	S	S	R	S	S	R	S	-	-	-	+	-	-	-	+	-	-	-	-	-
I11	S	S	S	S	S	S	S	S	S	S	-	-	-	-	-	-	-	+	-	-	-	-	-
I12	S	S	S	S	S	S	S	S	S	S	+	-	-	-	-	-	-	+	-	-	-	-	-
I13	S	I	S	S	S	S	R	S	S	S	-	-	-	-	-	-	-	+	+	-	-	-	+
I14	S	S	S	S	S	S	R	S	S	S	+	-	-	-	-	-	-	+	+	-	-	-	+
I15	R	R	S	S	S	R	S	S	S	S	-	+	-	+	-	-	-	+	-	-	-	-	-
I16	S	S	S	S	S	S	S	S	S	S	-	-	-	-	-	-	-	-	+	-	-	-	-
I17	R	R	S	S	S	R	S	S	S	S	-	+	-	+	-	-	-	+	-	-	-	-	-
I18	R	R	S	S	S	R	S	S	S	S	-	+	-	+	-	-	-	+	-	-	-	-	-
I19	S	S	S	S	S	S	S	S	S	S	-	-	-	-	-	-	-	-	-	-	-	-	-
I20	S	S	S	S	S	S	S	S	S	S	-	-	-	-	-	-	-	+	-	-	-	-	-
I21	S	S	S	S	S	S	S	S	S	S	-	-	-	-	-	-	-	+	-	-	-	-	-
I22	S	S	S	S	S	S	S	S	S	S	-	-	-	-	-	-	-	+	-	-	-	-	-
I23	S	S	S	S	S	S	S	S	S	S	-	-	-	-	-	-	-	+	-	-	-	-	-

R, resistant; S, susceptible; I, intermediate; +, positive in specific PCR; −, negative in specific PCR. FOX, cefoxitin; CIP, ciprofloxacin; CPT, ceftaroline; DOX, doxycycline; ERY, erythromycin; GEN, gentamicin; LZD, linezolid; MEM, meropenem; VAN, vancomycin. *blaZ*, penicillin resistance; *mecA* and *mecC*, oxacillin resistance; *ermA*, *ermB*, *ermC* and *msrA*, erythromycin resistance; *norA,* ciprofloxacin resistance; *tetK*, *tetL*, *tetM* and *tetO,* tetracycline resistance; *aac(6′)-aph(2)”*, gentamicin resistance

### Biofilm susceptibility tests

**Fig. 1:**
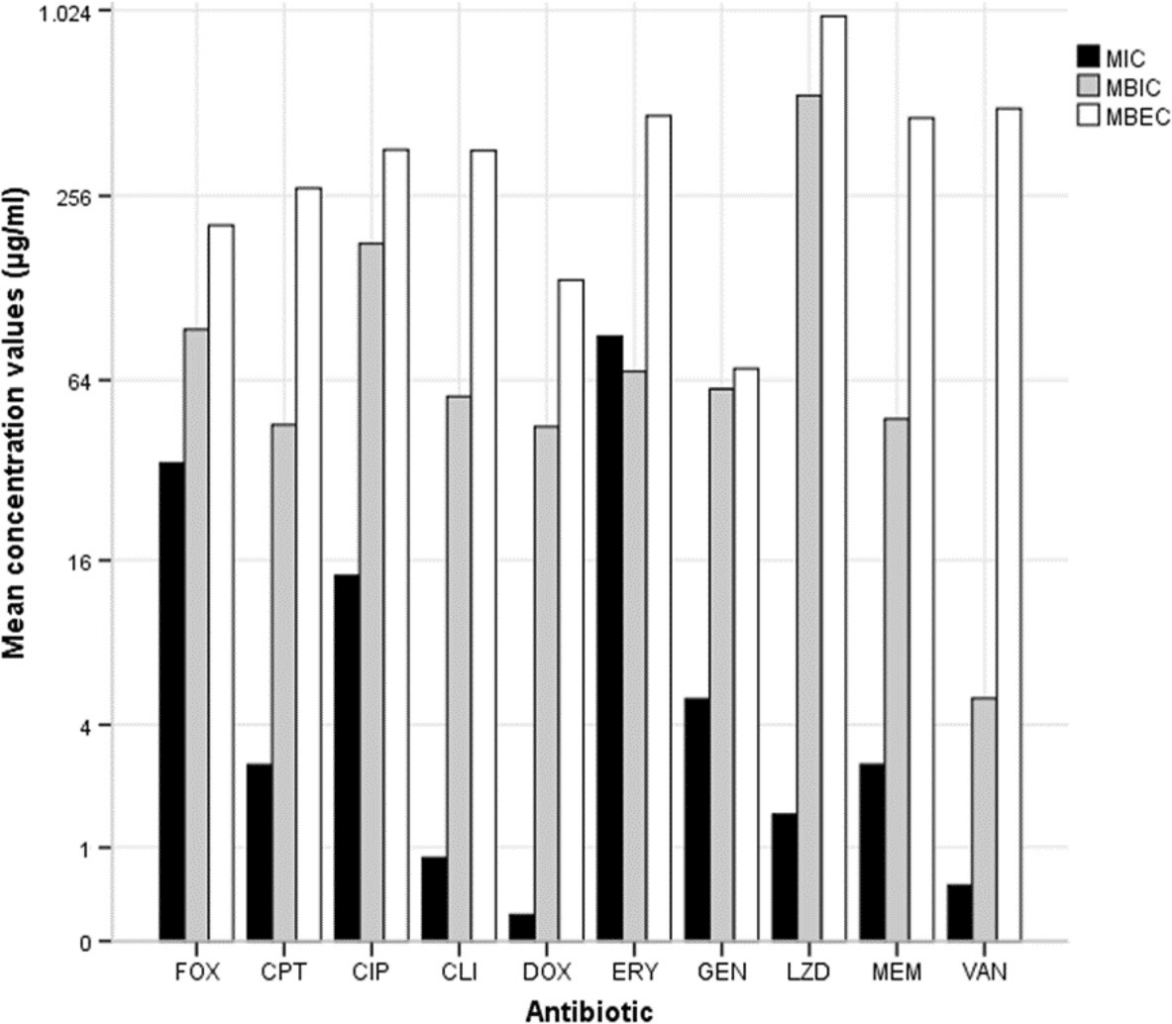
Minimum inhibitory concentration (MIC), minimum biofilm inhibitory concentration (MBIC) and minimum biofilm eradication concentration (MBEC) of *S. aureus* DFU isolates as determined by a modified version of the Calgary Biofilm Pin Lid Device. FOX, cefoxitin; CIP, ciprofloxacin; CPT, ceftaroline; DOX, doxycycline; ERY, erythromycin; GEN, gentamicin; LZD, linezolid; MEM, meropenem; VAN, vancomycin

MBIC and MBEC concentration values are summarized in Table 2. For MRSA isolates, cefoxitin MBIC concentrations ranged from two to five dilutions higher than MIC values, reaching values of 256 to 1024 μg/ml, while MBEC values were even higher (from 2 to ≥1024 μg/ml). Instead, for methicillin-susceptible *S. aureus* (MSSA) isolates MBIC and MBEC values for cefoxitin were the same as MIC values, with the exception of four isolates, for which MBIC and MBEC values were two times higher when compared to MIC. In 65 % of the isolates (*n* = 15), MBIC and MBEC values for linezolid were the same, and one thousand times higher when compared with MIC values (Fig. 1).

Other antibiotics originated different results. All MBIC and MBEC values were much higher than the respective MIC values, and MBEC values were at least twice higher than the MBIC ones. Ceftaroline MBIC values were four to sixteen times higher than MIC values, ranging from 0.5 to 8 μg/ml, except for one MRSA isolate that reached 1024 μg/ml; and the MBEC concentrations reached 1024 μg/ml, including four of the eight MRSA isolates. Regarding ciprofloxacin, MBIC values achieved an average of eight times higher compared with MIC, ranging from 0.5 to 512 μg/ml, except one isolate that reached 1024 μg/ml; for all isolates, except for two, MBEC values increased from 256 to 512 μg/ml. Similar results were obtained for clindamycin, but the gap between the MIC and MBIC values was higher, with MIC of 0.03 to MBIC of 0.5–128 μg/ml, except for one isolate that reached a MBIC value of 1024 μg/ml. MBIC values for doxycycline were even higher, as half of the isolates reached a MBIC concentration of 0.5 to 2 μg/ml, three hundreds to one thousand times higher than MIC. The other half showed MBIC values of 32 to 512 μg/ml, one thousand times higher than the respective MIC. Doxycycline MBEC values ranged from 64 to 128 μg/ml (Fig. 1).

The biofilm inhibition concentrations for erythromycin increased about four times in comparison with the values for MIC, from 0.12 - >256 μg/ml to 0.5 - >256 μg/ml. For MRSA, MIC and MBIC values were the same and equal to >256 μg/ml. These isolates reached MBEC concentrations of 64 - >1024 μg/ml, representing an increase of five hundred to one thousand times compared with MIC and MBIC values (except for the resistant isolates). In the case of gentamicin, it was observed an increase from MIC to MBIC of two to five hundred times higher, reaching values of 0.5 - >128 μg/ml (except for one MRSA isolate, that showed MBIC value of 1024 μg/ml); MBEC reached 256 μg/ml (including six of the eight MRSA isolates). Meropenem and vancomycin produced the major increase regarding MBEC values, being one thousand times higher than the value of MIC, and five hundred times higher than the MBIC values (0.5 to >1024 μg/ml and 8 to >1024 μg/ml for meropenem and vancomycin, respectively). The MBIC values ranged from 0.5 to 32 μg/ml for meropenem and from 1 to 16 μg/ml for vancomycin, except for one isolate that reached 1024 μg/ml (Fig. 1).

### PCR screening of biofilm associated and antibiotic resistance genes

All isolates were positive for the biofilm associated genes *icaA*, *icaD* and *atl,* and negative for *pls.* Eight isolates (35 %) were MRSA harbouring the *mecA* gene and were resistant to cefoxitin. None of the isolates presented the *mecC* gene. Three isolates presented the *blaZ* gene, one of which was MRSA and resistant to six of the antibiotics tested (Table 3).

Regarding the *tet* genes, none of the isolates were positive for *tetL* and *tetO*, one MRSA isolate was positive for *tetM* (and also *blaZ* positive), and *tetK* was found in three MSSA isolates. With the exception for two isolates, the erythromycin-resistant isolates were positive for *erm* genes, namely six for *ermA* (five MRSA and one MSSA) and three for *ermC* (two MRSA and one MSSA); contrariwise, none of these isolates was positive for *ermB* and *msrA* genes. The *aac(6′)-aph(2″)* gene was found in three isolates, one MRSA and two MSSA. Nineteen isolates (82 %) presented the *norA* gene, six of which were MRSA and thirteen were MSSA (Table 3).

## Discussion

The diversity of bacterial populations in chronic wounds, such as diabetic foot ulcers, and the biofilm mode of growth of the infecting organisms, may be important contributors to the chronicity of wounds [25]. As expected, all isolates carried the genes *icaA*, *icaD* and *atl*, due to their virulence profile and ability to form biofilm. None of the isolates were positive for *pls* gene, and this may suggest the adhesion and cellular invasiveness properties of the studied isolates, considering that the MRSA surface protein *pls* reduces these virulence features [13, 26].

Biofilm formation, as widely described in literature, represents a big obstacle for the clinical efficacy of antibiotics, and the results of antimicrobial susceptibility testing cannot be directly applied to bacterial biofilm infections, due to higher probability of failure [27]. Biofilm can resist even to antibiotic concentrations 10–10.000 times higher than the ones needed to kill planktonic cells [10]. In this study, antibiotic concentrations required to inhibit or eradicate biofilm were much higher than the respective MIC values and should not be clinically applied. Furthermore, MBEC values were often several times higher than MBIC values.

Although all MRSA isolates should be considered as resistant to β-lactams in vivo [15], almost all isolates were susceptible to meropenem. MBIC values for this antibiotic were thirty to one thousand times higher than MIC, being still in the range of susceptibility, however meropenem was unable to eradicate biofilm. The results suggested that cefoxitin is able to inhibit and eradicate *S. aureus* biofilms formed by MSSA isolates.

PCR amplification of *mecA* is considered the “gold standard” technique for detection of methicillin resistance among *S. aureus* [28]. However, the discovery of a new *mecA* homologous gene, *mecC*, determined the need to establish new detection protocols [24], although normally the screening of the homologous gene is only performed in oxacillin-resistant *mecA* negative isolates [29]. In this study, a multiplex assay was applied for the screening of *mecA* and *mecC* in all isolates, being possible to detect the *mecA* gene in 35 % of the *S. aureus* DFU isolates (*n* = 8). By the contrary, *mecC* was not detected in any isolates, which is not surprising because MRSA isolates harbouring *mecC* are currently rare, and have only been reported in 13 European countries to date, not including Portugal [30]. The presence of *mecA* positive strains among the study isolates can be associated with the increasing prevalence of antibiotic-resistant bacteria in DFU isolates, particularly MRSA, as described by Bowling et al. [31]. Also, Djahmi et al. suggested that MRSA prevalence may be related with the increase of antimicrobial treatment required, considering the high frequency of recurrent ulcers [32]. Nowadays, penicillin resistance is present in about 90 % of human *S. aureus* isolates. Two mechanisms are involved: the production of β-lactamases encoded by the *blaZ* gene and an altered penicillin-binding protein, PBP2a, encoded by *mecA* [33, 34]. In our study, only three isolates were positive for *blaZ*. This may be due to the primers used, because multiple polymorphisms within the *blaZ* gene have already been identified and the results can vary when different regions of the gene are targeted [34], or may also be due to the fact that the isolates express penicillin resistance encoded by *mecA*.

Antibiotic susceptibility tests showed that gentamicin and ceftaroline were the most potent agents against *S. aureus* biofilms, reaching clinical concentrations that can be applied to inhibit and eradicate biofilms. This was observed even for the MRSA isolates, since ceftaroline and gentamicin were effective in inhibiting biofilm production by seven of the eight MRSA isolates, while ceftaroline was effective in eradicating biofilm production by half of the MRSA isolates and gentamicin was effective in eradicating biofilm production by two isolates MRSA isolates. Today ceftaroline represents a good alternative to treat infections by *S. aureus* with reduced susceptibility to current agents, as recent studies have proven its efficacy against biofilm, applied alone or in combination [35, 36].

In 1999, Ceri et al. [16] already described the efficacy of gentamicin against *S. aureus* biofilms, as well as other studies that followed [20, 37]. The *aac(6′)-aph(2'')* is the gene coding for the most frequently encountered aminoglycoside modifying enzyme (AME) in Gram-positive bacteria, which inactivates a broad range of clinically useful aminoglycosides, especially gentamicin and tobramycin; this enzyme is bifunctional because it catalyses both acetyltransferase and phosphotransferase reactions [38]. The *aac(6′)-aph(2'')* gene was found in three isolates, one MRSA and two MSSA, with no discrepant results with the resistance phenotypes obtained by e-test. These findings are in agreement with other studies, which reported that all aminoglycoside-resistant strains carried the *aac(6′)-aph(2'')* gene [38].

Linezolid lacked activity against staphylococci biofilms because it didn’t inhibit or eradicate biofilms, as already reported in other studies [18, 20, 39]. Clindamycin, doxycycline and vancomycin were effective against planktonic cultures and inhibited biofilm produced by most isolates; however, these antibiotics showed no ability to eradicate biofilms. This may suggest that these agents, although effective against bacteria in suspensions, may not be the most suitable antibiotics for treating biofilm related infections. Previous studies have shown that these antibiotics lack activity against staphylococci grown in biofilms [17–19, 39, 40].

Although isolates presented a high rate of resistance to ciprofloxacin and erythromycin, their MBIC concentrations were about eight times higher than MIC values but were still clinically adequate. They were not able to eradicate biofilm, as previously described for ciprofloxacin in other studies [16, 18]. Several genes are implicated in erythromycin resistance, especially in staphylococci and streptococci. The gene *ermA* is located on the transposon Tn554 and has a single specific site for insertion into *S. aureus* chromosome; the *ermB* gene is located on the transposon Tn551 of a penicillinase plasmid; the *ermC* gene is generally located on small plasmids and is responsible for constitutive or inducible resistance to erythromycin [38]. Staphylococcal strains resistant to macrolides and type-B streptogramins also frequently harbor *msrA,* which encodes an ATP-dependent efflux pump [38]. In this study, *ermA* was found in five resistant and one sensible erythromycin staphylococci. Regarding *ermC*, this gene was less frequently found than *ermA,* namely in two resistant and one sensible erythromycin staphylococci. These results are according to previous reports, in which *ermA* was the most prevalent *erm*-gene followed by *ermC* [38, 41, 42]. The discrepance between the erythromycin susceptible isolates and their *erm* positivity was already described [38]. Numerous factors could explain the sensitive phenotype in these strains, including regulation of *erm* genes and absence of host factors associated with the expression of erythromycin resistance. These factors can also explain the cefoxitin-susceptible *S. aureus* isolates carrying the *mecA* gene.

The increasing prevalence of MRSA has led to a new interest in the usage of macrolide-lincosamide-streptogramin B (MLS_B_) antibiotics to treat *S. aureus* infections, with clindamycin being the preferred agent due to its excellent pharmacokinetics. However, this increased application promoted a raise in resistance to MLS_B_ antibiotics. Clindamycin resistance is commonly caused by a one target site modification mediated by *erm* genes, difficult to detect in vitro, as they appear erythromycin resistant and clindamycin sensitive [43].

Tetracycline resistance determinants are widespread among bacterial species, consisting in active efflux pumps that result from acquisition of plasmid-located genes, *tetK* and *tetL*, and in ribosomal protection mediated by transposon or chromosomal located genes *tetM* or *tetO* [30, 42]. MRSA isolates typically show *tetM* or *tetKM* genotype; *tetK* is the most frequent genotype found in *S. aureus*, followed by *tetM* [39, 43, 44]. The same was observed in this study, in which three MSSA isolates were *tetK* positive and one MRSA was *tetM* positive; *tetL* and *tetO* were not found. *Tet*-positive isolates were sensible to doxycycline. In *tetK* positive isolates, this gene confers high resistance to tetracycline, oxytatracycline, chlortetracycline but low resistance to monocycline, 6-demethyl-6-deoxytetracycline and doxycycline [44]. Surprisingly, the only *tetM* positive isolate was doxycycline sensible. Since this gene is believed to confer resistance to all drugs of tetracycline group [45], it may be suggested that prevalence of resistance to tetracyclines in *S. aureus* is underestimated or, as demonstrated by Trzcinski et al. [45], recognition of tetracycline resistance in *S. aureus* strains often depends on the different interpretation guidelines used.

In recent years, an increase in fluoroquinolone resistance in *S. aureus*, including MIC strains, has been spreading worldwide. Resistance mechanisms to these antibiotics involve mutations within the *gyrA* and *gyrB* genes, which encode for subunits of DNA gyrase, an established target of fluoroquinolones; analogous mutations in *grlA* and *grlB*, which encode for subunits of DNA topoisomerase IV; and the increased expression of the *norA* gene, which encodes for a drug efflux protein, *norA*, and mutations in the *norA* coding region [45, 46]. *NorA* is a membrane protein which actively transports norfloxacin and other hydrophilic fluoroquinolones out of the bacterial cell, thus effectively decreasing the intracellular concentration of the drugs [45]. Nineteen isolates were positive for *norA* gene but eleven of them were susceptible to ciprofloxacin.

From a clinical perspective, the discrepancy between genotype and phenotypic resistance expression suggest that a susceptible strain harbouring, but not expressing, an antibiotic resistance gene should be regarded as potentially resistant to that antibiotic. Overall, we did not detect a significant presence of antibiotic resistance genes, compared to the great biofilm resistance of the isolates, even when using high antimicrobial concentrations.

## Conclusions

To our knowledge, this is the first study on antibiotic susceptibility tests targeting biofilm-producing *S. aureus* isolates from diabetic foot infections. It was found that only very high concentrations of the most used antibiotics in DFU were capable to inhibit *S. aureus* biofilms in vitro, which may explain why monotherapeutics frequently fail to eradicate biofilm infections. Biofilms were resistant to antibiotics concentrations 10 to 1000 times higher than the concentrations needed to kill free-living or planktonic cells. This high level of resistance in biofilms makes chronic infections, like DFI, extremely difficult to eradicate using conventional antimicrobial therapy.

MIC values were not always predictive of the MBIC and MBEC values. Only gentamicin and ceftaroline proved to be effective in eradicating biofilms, formed by half of isolates at clinical drug concentrations, while the other tested drugs were only able to inhibit adherent cells. In particular, ceftaroline showed a very good potential for inhibiting and eradicating biofilms produced by MRSA isolates. It is clear that antibiotic susceptibility values for planktonic populations are not necessarily applicable to effective treatment of infections by the same organism, once a biofilm has been established. These differences may be an important factor in the selection of antimicrobial therapy for most of DFI, to whom *S. aureus* is the main virulent organism involved, rendering important the investigation of antibiotic susceptibility of biofilm infections.

## Abbreviations

*atl*, autolysin; DFI, diabetic foot infections; DFU, diabetic foot ulcers; *icaA*, intercellular adhesion *A*; *icaD*, intercellular adhesion *D; icaR*, intercellular adhesion regulator; MBEC, minimum biofilm eradication concentration; MBIC, minimum biofilm inhibitory concentration; MIC, minimum inhibitory concentration; MRSA, methicillin-resistant *S. aureus*; MSSA, methicillin-susceptible *S. aureus*; PBP2a, penicillin binding protein 2a; *pls*, plasmin sensitive
